# Mobilization of Stem and Progenitor Cells in Septic Shock Patients

**DOI:** 10.1038/s41598-019-39772-4

**Published:** 2019-03-01

**Authors:** Tomasz Skirecki, Małgorzata Mikaszewska-Sokolewicz, Marlena Godlewska, Barbara Dołęgowska, Jarosław Czubak, Grażyna Hoser, Jerzy Kawiak, Urszula Zielińska-Borkowska

**Affiliations:** 1 Department of Anesthesiology and Intensive Care Medicine, Centre of Postgraduate Medical Education, Marymoncka 99/103, 01-813 Warsaw, Poland; 2 Laboratory of Flow Cytometry, Centre of Postgraduate Medical Education, Marymoncka 99/103, 01-813 Warsaw, Poland; 3 Nalecz Institute of Biocybernetics and Biomedical Engineering, PAS, Trojdena 4, 02-109 Warsaw, Poland; 4 The 1st Clinic of Anesthesiology and Intensive Care Medicine, Medical University of Warsaw, Lindelya 4, 00-001 Warsaw, Poland; 5 Department Biochemistry and Molecular Biology, Centre of Postgraduate Medical Education, Marymoncka 99/103, 01-813 Warsaw, Poland; 6 Department of Laboratory Medicine, Pomeranian Medical University, Powstańców Wlkp. 72, 70-111 Szczecin, Poland; 7 Department of Pediatric Orthopedics, Centre of Postgraduate Medical Education, Marymoncka 99/103, 01-813 Warsaw, Poland

## Abstract

Septic shock is associated with multiple injuries to organs and tissues. These events may induce the regenerative response of adult stem cells. However, little is known about how endogenous stem cells are modulated by sepsis. This study analyzed the circulation of hematopoietic stem cells (HSCs), endothelial progenitor cells (EPCs) and very small embryonic-like stem cells (VSELs) in the peripheral blood of patients with septic shock. Thirty-three patients with septic shock and twenty-two healthy control subjects were enrolled in this prospective observational study. Blood samples were collected on the first, third and seventh days of septic shock. Populations of stem cells were analyzed by flow cytometry. Chemotactic mediators were analyzed by HPLC and ELISA. Populations of early HSCs (Lin-CD133+CD45+ and CD34+CD38−) were mobilized to the peripheral blood after an initial decrease. Mobilized HSCs showed significantly increased expression of Ki-67, a marker of cell proliferation. Circulating EPCs and VSELs were mobilized to the blood circulation upon the first day of sepsis. Patients with a greater number of Lin-CD133+CD45+ HSCs and Lin-CD34+CD45− VSELs had a significantly lower probability of 60-day survival. The concentration of CXCL12 was elevated in the blood of septic patients, while the concentration of sphingosine-1-phosphate was significantly decreased. As an emergency early response to sepsis, VSELs and EPCs were mobilized to the peripheral blood, while the HSCs showed delayed mobilization. Differential mobilization of stem cell subsets reflected changes in the concentration of chemoattractants in the blood. The relationship between the probability of death and a large number of HSCs and VSELs in septic shock patients can be used as a novel prognostic marker and may provide new therapeutic approaches.

## Introduction

Sepsis is a leading cause of death in the US and Europe^1^. It remains a resource-consuming condition with high mortality, although no specific treatment has been implemented thus far. The inflammatory-driven maladaptive response induces endothelial and epithelial barrier disruption resulting in organ dysfunction^2^. The alleviation of these disturbances with subsequent regeneration is a prerequisite for recovery and survival.

Adult organisms contain a variety of stem and progenitor cells that are responsible for the continuous renewal and regeneration of damaged tissues. Bone marrow hematopoietic stem cells (HSCs) and hematopoietic progenitor cells (HPCs) are responsible for maintaining adult hematopoiesis^3^. The host response to infection induces the activation and proliferation of HSCs, which is referred to as emergency myelopoiesis^3^. However, studies in animal models have shown that sepsis influences hematopoiesis by stimulating the proliferation of HSCs with concomitant induction of functional impairment^4–6^. Whether dysfunctional immune responses in septic patients are caused by impaired hematopoiesis remain unknown. Although, under physiological conditions, only a few stem cells have been observed in peripheral blood^7^; however, under stress conditions, numerous stem cells have been shown to migrate into the blood circulation^8^. The circulation of HSCs is strictly regulated by molecular interactions responsible for their retention in bone marrow (i.e., CXCL12-CXCR4) and other interactions that orchestrate their mobilization into the blood (sphingosine phosphate (S1P) gradient – SP1R)^9^. It is hypothesized that sepsis affects these regulatory axes. Aside from HSCs, bone marrow contains other stem cell populations, such as endothelial progenitor cells (EPCs) and primitive very small embryonic-like stem cells (VSELs), which have the potential to differentiate into multiple cell types, including hematopoietic cells^10–12^. VSELs constitute a rare population of pluripotent/multipotent quiescent adult stem cells that express transcription factors related to pluripotency^13^. Due to the specific imprinting pattern of the insulin growth factor signaling genes, these cells are activated in a strictly regulated manner and have been hypothesized to contribute to the tissue renewal and regeneration^13^. The mobilization of HSCs, EPCs and VSELs has been reported in several clinical conditions (myocardial infarction, cerebral ischemia, and severe burns) and has been shown to be related to the outcome of these conditions^14–17^. To date, in septic patients, only circulating endothelial progenitor cells (cEPCs) have been investigated, and the number of cEPCs has been shown to be correlated with survival^18^. It is speculated that the mobilized stem cells can contribute to the regeneration of injured tissues and enhance the immune response via direct and paracrine mechanisms.

There is limited information regarding the influence of sepsis on the circulation and mobilization of HSCs, HPCs and VSELs. Examining the influence of sepsis on the circulation and mobilization of these cells could improve the understanding of the extent of perturbation of stem cell homeostasis in sepsis and the role of stem cells in the pathogenesis of sepsis. Therefore, we conducted a study aimed at evaluating the circulation of different stem cell populations during septic shock.

## Methods

### Patient recruitment

This prospective observational study enrolled septic shock patients treated in the general ICUs of two teaching hospital (Professor Orlowski Independent Public Clinical Hospital and The Infant Jesus Teaching Hospital in Warsaw) in the period between March 2012 and January 2014. This study was approved by the Centre of Postgraduate Medical Education Ethics Board in accordance with the Helsinki Declaration. Informed consent was obtained from patients in agreement with the rules of the Ethics Committee. Patients with suspected or proven infection who fulfilled the septic shock criteria according to the 2001 ACCP/SCCM Consensus Conference^19^ definitions were enrolled in this study within 24 h (from the beginning of vasopressor treatment), and informed consent was obtained in agreement with the rules of the Ethics Committee. The exclusion criteria included: ages <16 or > 85y.o., immunosuppressive treatment, hematological malignancy, metastatic neoplasms, HIV infection, and pregnancy. All patients received standard treatment recommended by the Surviving Sepsis Guidelines, 2008^20^. Standard clinical data were recorded. The healthy control group consisted of sex- (11 male) and age-matched (median: 63 (IQRs: 50–78)) volunteers with no chronic diseases who had no symptoms of infection.

Bone marrow for the chemotaxis assay was collected during elective hip surgery from adult patients with no chronic inflammatory disease who provided informed consent (Gruca Orthopedic and Trauma Teaching Hospital, Otwock, Poland).

### Flow cytometry (FACS)

The total cell count was analyzed using the Burk’s hemocytometer and the Turk’s solution. Immunocytochemical staining was performed as previously described^21^. Briefly, 100 μl of peripheral blood (PB) was stained with cocktails of antibodies (Table S1.) and incubated for 30 min. at room temperature. Then, cells were lysed with BD Pharm Lyse (BD Biosciences, San Jose, USA) for 10 min, washed and fixed in 0.5% paraformaldehyde (Sigma-Aldrich, St. Louis, USA). For intracellular staining of Ki-67, fixed cells were washed with PBS and permeabilized with 0.25% Triton X-100 (Sigma-Aldrich) in PBS for 10 min and labeled with antibodies for 30 min. Appropriate isotype and FMO (fluorescence minus one) controls were used. The cells were analyzed using a FACS Canto II flow cytometer with FACS Diva software (BD Bioscience). At least 100,000 events were recorded in the mononuclear cell gate set on the SSC/FSC morphological plot. The results were analyzed using FlowJo V10 software (Tree Star Inc, Ashland, USA).

### CXCL12 and S1P concentrations

The concentration of circulating CXCL12 was measured in low-platelet plasma using a commercially available ELISA kit (R&D Systems, Minneapolis, USA) according to the manufacturer’s protocol.

The concentration of S1P in plasma was measured using high-performance liquid chromatography (HPLC) method as described previously^22^, and the detailed protocol is included in the supplemental methods.

### Chemotaxis assay

Migration of nucleated bone marrow cells towards human plasma was assessed using a Transwell migration assay. Nucleated bone marrow cells were isolated from bone marrow samples after erythrocyte lysis. A total of 650 μl of RPMI medium (Gibco, Thermo Fisher, Rochester, USA) containing 10% of plasma from septic patients (pooled from 10 patients on day 1) or pooled normal human plasma (obtained from 10 healthy volunteers) was placed in the lower chamber of a 24-well plate equipped with a 3 μm ThinCert (Greiner Bio-One, Kremsmünster, Austria) Transwell insert. Then, 1 × 10^6^ nucleated bone marrow cells suspended in 100 μl of RPMI medium were loaded into the upper chamber, and the plate was incubated at 37 °C and 5% CO_2_ for 2.5 h. Cells that migrated to the lower chamber were collected and stained with anti-CD34 PE and anti-CD38 PE.Cy5 monoclonal antibodies (BD Biosciences) and counted using a flow cytometer (FACS Canto II, BD Biosciences). In some experiments bone marrow cells were preincubated in 100 nM sphingosine-1 phosphate (Cayman Chemical, Michigan, USA) for 60 minutes before chemotaxis assay. In total, experiments were performed with cells from five bone marrow donors. Due to the very low number of VSELs, we did not perform these assays for VSELs.

### Statistical analysis

The absolute number of cells per milliliter of PB was calculated from white blood counts and percentage frequencies from flow cytometry analyses. Data are expressed as medians and interquartile range values. Comparisons between the control and septic groups were performed by Mann-Whitney U test. For the comparison of the cell number between days 1 and 3, the Wilcoxon signed-rank test was used. The strength of the correlation between two variables was assessed using the Spearman’s rank correlation coefficient. Logistic regression was performed to assess the odds ratio of death depending on the number of circulating stem cells. Receiver operator curves (ROC) were calculated for the evaluation of the predictive utility of analyzed stem cell populations. Cut-off values were chosen using Youden’s method after the ROC analysis was conducted to stratify patients with high and low numbers of circulating populations of particular of cells. Then, Kaplan-Meier survival curves were compared using Cox’s F-test. In migration experiments Anova with Dunnet’s multiple comparison test was used. p < 0.05 was considered statistically significant. Statistica 12 (StatSoft, Inc., USA) and GraphPad Prism 5 (GraphPad, Inc., USA) software were used.

### Ethics approval and consent to participate

The Centre of Postgraduate Medical Education Institutional Review Board approved this study. All patients and healthy volunteers who met the eligibility criteria were consented prior to blood sampling.

## Results

### Patient characteristics

For this study, 33 patients were enrolled based on the inclusion and exclusion criteria. A summary of the clinical data is presented in Table 1. Patients who died had increased APACHE II scores (26 vs. 20, p < 0.001), SOFA scores (12 vs. 8, p < 0.001), and SAPS scores (63 vs. 43, p < 0.001) compared with patients who did not die during the observation period.

**Table 1:** Demographic and clinical characteristics of the studied septic shock patients.

	N (%)	Median (IQR)
Age		65 (53–77)
Sex (M)	16 (48%)	
Hospital mortality	19 (57%)	
7-day mortality	5 (15%)	
Infection source		
peritoneum	20 (60.6%)	
lungs	10 (30.3%)	
other	3 (9.1%)	
Positive blood cultures Gram positive/negative	19 (57%)	
	5/14	
APACHE II		22 (20–31)
SAPS		57 (44–65)
SOFA D1		11 (8–12.5)
Lactate D1 (mg/dl)		24 (18–38.2)
CRP D1 (mg/l)		207 (132–357)
PCT D1 (ng/ml)		6.9 (2.7–39)
ARDS	14 (42%)	

APACHE II: Acute Physiology and Chronic Health Evaluation II; ARDS: acute respiratory distress syndrome; CRP: C-reactive protein; IQR: interquartile range; PCT: procalcitonin; SAPS: simplified acute physiology score; SOFA: sequential organ failure assessment.

### Circulation of hematopoietic and VSEL stem cells

**Figure 1:**
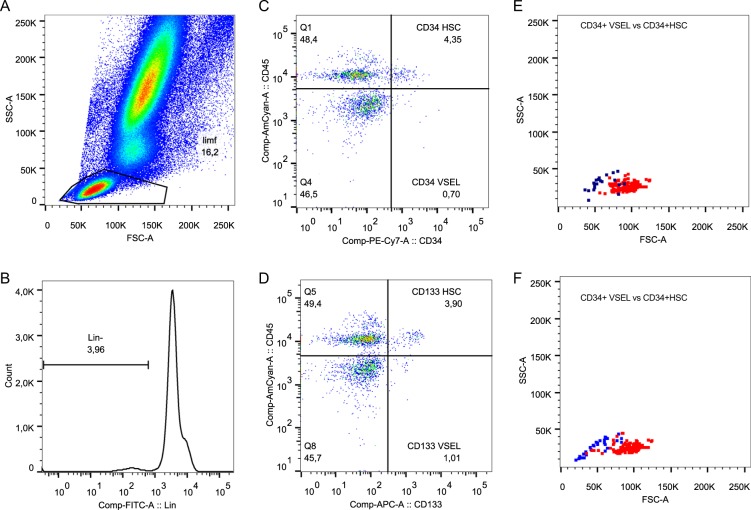
Representative gating strategy for the analysis of VSELs and HSCs. (**A**) First, an extended lymphocyte gate was created, (**B**) then cells negative for the Lineage cocktail antigens are gated. (**C**) Lin- cells were then analyzed for the expression of CD45 and CD34 and (**D**) CD45 and CD133. (**E**) Lin-CD34+CD45+ HSCs and Lin-CD34+CD45− VSELs were then plotted on the SSC/FSC dot plot to compare their granularity and size. (**F**) SSC/FSC dot plot graph showing Lin-CD133+CD45− VSELs and Lin-CD133+CD45+ HSCs.

**Figure 2:**
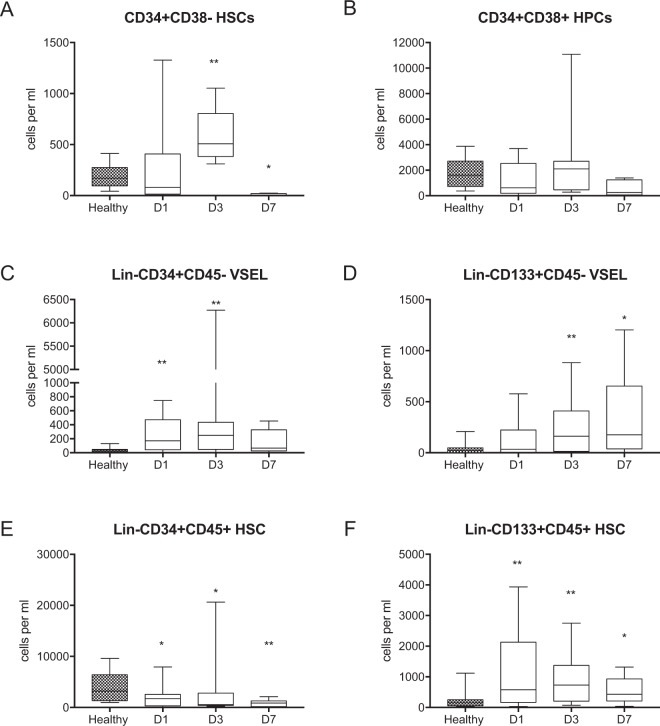
Total numbers of circulating stem cell subpopulations per mL of peripheral blood. (**A**) CD34+CD38– HSCs. (**B**) CD34+CD38+ HPCs. (**C**) Lin-CD34+CD45− VSELs. (**D**) Lin-CD133+CD45− VSELs. (**E**) Lin-CD34+CD45+ HSCs. (**F**) Lin-CD133+CD45+ HSCs. Comparisons between the control and septic groups were performed by Mann-Whitney U test. Statistical significance between values for healthy controls and septic shock patients are marked as follows: *p < 0.05 and **p < 0.01. The comparison of the cell number between days 1 and 3 by the Wilcoxon signed-rank test did not reveal significant differences.

To investigate the kinetics of stem cell circulation, we focused on the analysis of the absolute counts of specific cell populations. We used different combinations of antibodies specific for hematopoietic stem cell markers, which enabled the use of several strategies for stem cell identification **(**Fig. 1**)**. Because the CD34+CD38− phenotype has been extensively used to identify HSCs in numerous studies, we included this phenotype in our panel (Supplemental Fig. 1). The frequency of circulating CD34+CD38− HSCs was significantly decreased on the days 1 and 7 of sepsis compared to the healthy controls (Supplemental Table 2**)**. Regarding absolute values, on day 3 the number of CD34+CD38− HSCs was 3-fold higher than in healthy controls (Fig. 2A). However, there were no significant changes in the number of circulating CD34+CD38+ progenitor cells (Fig. 2B, Supplemental Table 2). The proper identification of VSEL stem cells requires strict gating criteria, which results in the ability to observe and analyze this rare population of CD45-negative stem cells that are smaller than the CD45+ HSCs (Fig. 1A–D)^23^. This morphological difference is shown in the SSC/FCS dot plots, which show VSELs vs. HSCs (Fig. 1E,F). Both stem cell populations have similar granularity, but VSELs are less abundant than HSCs. Additionally, both populations are Lin- (e.g., they are negative for: CD19, CD2, CD3, CD4, CD14, CD66b, CD24, CD16, CD56 and CD235a). The number of circulating Lin-CD34+CD45− VSELs was 4-fold greater on day 1 of sepsis than that in healthy controls, and the number of circulating Lin-CD34+CD451− VSELs was the greatest at day 3 (Fig. 2C, Supplemental Table 2). Similarly, Lin-CD133+CD45− VSELs migrated to the peripheral blood upon the onset of sepsis, and their number remained elevated on day 7 (Fig. 2D, Supplemental Table 2). The number of HSCs identified as Lin-CD34+CD45+ in the circulation of septic shock patients was significantly less than that in the healthy controls (Fig. 2E, Supplemental Table 2), while HSCs gated as Lin-CD133+CD45+ cells migrated to the peripheral blood on day 1 (Fig. 2F, Supplemental Table 2). However, neither of the HSC populations differed significantly between the analyzed timepoints of sepsis.

### Circulation of endothelial progenitor cells

**Figure 3:**
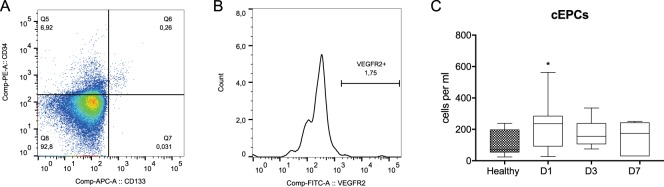
Analysis of the circulating endothelial cells. (**A**) A cytogram of CD34 versus CD133 expression gated from the extended lymphocyte gate (Fig. 1A). (**B**) VEGFR2 receptor expression by double-positive progenitor cells. (**C**) Dynamics of the changes in the total number of cEPCs in septic shock patients. Cell counts between healthy controls and septic shock patients were compared using Mann-Whitney U test and the statistical significance was marked as follows: *p < 0.05. Cell counts between days 1 and 3 was compared by the Wilcoxon signed-rank test which did not reveal significant differences.

Additionally, we determined the number of cEPCs because their mobilization in sepsis has been previously reported^18^. We gated cEPCs as CD34+CD133+VEGFR2+ mononuclear cells (Fig. 3A,B). The number of cEPCs was significantly greater in septic patients only on day 1 compared to that in healthy controls (Fig. 3C, Supplemental Table 2).

### Proliferation of circulating hematopoietic stem and progenitor cells (HSPCs) in septic shock patients

**Figure 4:**
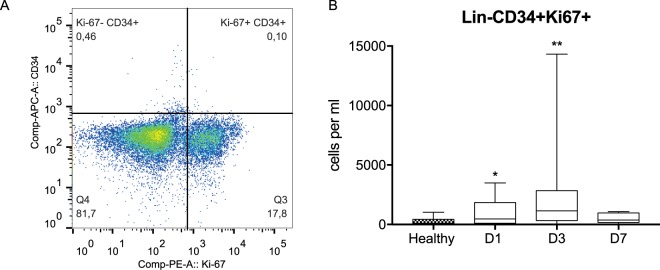
Analysis of the proliferation of circulating hematopoietic stem and progenitor cells by the expression of Ki-67. (**A**) Lineage-negative cells (Fig. 1A,B) were analyzed for the expression of the Ki-67 antigen in the CD34+ cells. (**B**) The dynamics of the circulation of Lin-CD34+Ki-67+ cells in septic shock patients. Cell counts between healthy controls and septic shock patients were compared using Mann-Whitney U test and the statistical significance was marked as follows: *p < 0.05. Cell counts between days 1 and 3 was compared by the Wilcoxon signed-rank test which did not reveal significant differences.

We also aimed to investigate whether the mobilized HSPCs are more proliferative than HSPCs under physiological conditions. Therefore, samples were stained for HSPC markers and the Ki-67 antigen. HSPCs positive for Ki-67 expression were observed in control subjects and patients with sepsis (Fig. 4A). On day 1 of sepsis, the number of Lin-CD34+Ki-67+ cells rapidly increased compared to the healthy volunteers (469 (109–1578) cells per ml vs. 105 (47–375) cells per ml, respectively; p < 0.05). An increase was observed on day 3 (1148 (421–2750) cells per ml), while on day 7, the number of proliferating HSPCs began to decrease (367 (164–965) cells per ml) (Fig. 4B). Interestingly, we found a significant correlation between the number of Lin-CD34+Ki-67+ cells and the C-reactive protein (CRP) concentration on day 3 (r = 0.9, p < 0.05).

### Stem cell counts and clinical variables

**Figure 5:**
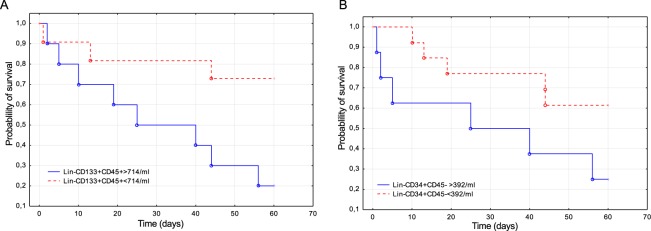
Probability of 60-day survival of septic shock patients in relation to the number of circulating stem cells. (**A**) Survival curves of patients with varied number of Lin-CD133+CD45+ HSCs. (**B**) Survival curves of patients with varied numbers of Lin-CD34+CD45− VSELs.

There was no relationship between the stem cell population counts and the patient sex, age or source of infection (data not shown). The frequencies of circulating Lin-CD34+CD45− and Lin-CD133+CD45− VSELs on day 1 was correlated with the SOFA score (r = 0.56 and r = 0.44, respectively, p < 0.05). There was also a negative correlation between the frequency of VSELs and the PaO_2_/FiO_2_ ratio on day 1 (r = −0.44, p < 0.05 for both Lin-CD34+CD45− and Lin-CD133+CD45− VSELs). Logistic regression analysis did not reveal significant relationships between mortality from sepsis and the median numbers of circulating stem cell populations. Additionally, we evaluated the potential of circulating stem cells as outcome predictors by performing ROC analysis. The two stem cell populations with the greatest AUC were Lin-CD34+CD45− VSELs and Lin-CD133+CD45+ HSCs (0.72 95% CI (0.50–0.94) and 0.67 95% CI (0.41–0.93), respectively). Then, we compared the patient survival curves based on the number of VSELs and HSCs selected by the Youden’s method. Septic shock patients who had less than 392 Lin-CD34+CD45− VSELs per ml of blood had a greater probability of survival (p = 0.03). Patients who had less than 714 Lin-CD133+CD45+ HSCs per ml of blood were more likely to survive (p = 0.005) (Fig. 5).

### Mechanisms of HSPC mobilization in sepsis

**Figure 6:**
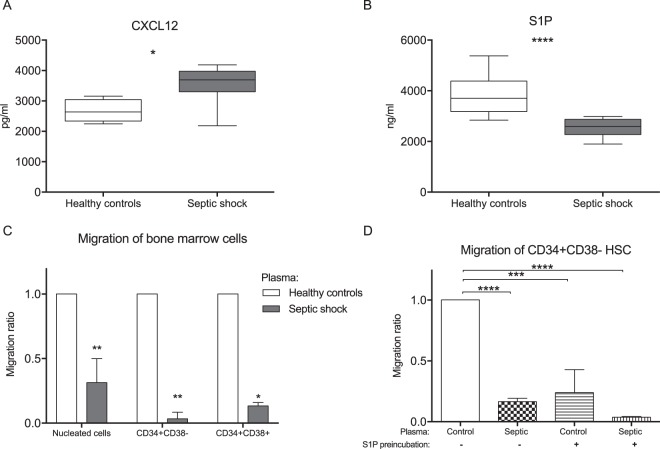
Mechanisms of stem cell mobilization. (**A**) Serum levels of CXCL12 in septic shock patients and healthy volunteers. (**B**) Plasma concentrations of S1P in septic shock and in controls. (**C**) The chemotactic activity of septic plasma was evaluated using a Transwell migration assay on human bone marrow cells. The migration of all nucleated cells, CD34+CD38− HSCs and CD34+CD38+ HPCs was analyzed. The results were normalized to the number of cells that migrated to the normal human plasma (n = 5). (**D**) Migration of bone marrow cells that were preincubated in 100 nM S1P or medium only for 60 minutes before the chemotaxis assay. The concentration of mediators was compared using Mann-Whitney U test (**A**,**B**) and comparisons between multiple groups were performed using Anova with Dunnet’s multiple comparison test (**C**,**D**) *p < 0.05, **p < 0.01, ***p < 0.001 and ****p < 0.0001.

To investigate the mechanism of HSPC dynamic circulation during sepsis, we first analyzed the two well-known mediators of chemotaxis for HSPCs, CXCL12 and S1P. The plasma level of the CXCL12 was significantly greater in septic shock patients on day 1 compared to the healthy controls (Fig. 6A). In contrast, the plasma level of S1P was lower in septic patients compared to the healthy controls (Fig. 6B). Then, we evaluated the *ex vivo* chemotactic activity of plasma from septic patients. Plasma from septic patients demonstrated much less chemotactic activity than control plasma. This was observed for all nucleated bone marrow cells and, in particular, CD34+CD38− HSCs and CD34+CD38+ HPCs (0.31 and 0.03, respectively, and 0.13 for normal plasma activity; Fig. 6C). In order to verify the involvement of S1P and its receptors in the migration of HSCs to plasma, we incubated bone marrow cells in 100 nM S1P to induce internalization of S1P receptors^24^. Preincubation of bone marrow cells with S1P decreased CD34+CD38− HSCs migration to normal plasma to the level comparable with migration of untreated bone marrow cells towards septic plasma (Fig. 6D).

## Discussion

In this study, we found that during septic shock, a differential kinetics characterize stem cell populations with early mobilization of primitive VSELs and cEPCs and enhanced mobilization of proliferating HSCs. Importantly, increased mobilization of primitive stem cells was correlated with unfavorable outcomes.

We found that CD34+CD38− HSCs, which are known to contain all repopulating cells^25^, migrated to the peripheral blood on day 3. Similar kinetics characterized the circulation of Lin-CD34+CD45+ HSCs, which contain also more mature progenitor cells. Only the rare population of primitive Lin-CD133+CD45+ HSCs was mobilized at day 1 and remained elevated. To the best of our knowledge, the only report on hematopoietic progenitor cells in sepsis has been published by Tsaganos *et al*.^26^. In their study, only the population of CD34+CD45+ cells was increased in septic patients. Cells of this phenotype also contain committed myeloid and lymphoid progenitors^27^, which makes it impossible to compare their results with our current work. Similar to our work, Drukala *et al*.^17^ found a decreased number of circulating Lin-CD34+CD45+ HSCs and Lin-CD133+CD45+ HSCs at early timepoints in severely burned patients^17^. Interestingly, we found that the mobilized HSPCs are actively proliferating. This result is consistent with our previously reported results showing the enhanced proliferation of human HSCs in the bone marrow of humanized mice after cecal ligation and puncture sepsis^4^. A strong correlation between the number of proliferating HSCs and CRP levels supports the link between inflammation and activation of otherwise dormant HSCs. Observations from other acute conditions that are associated with HSC mobilization^14,28^ suggest that this process is part of the stress response and may be protective. Although the major function of HSCs is the maintenance of central hematopoiesis, mounting evidence has indicated that these cells are active players in the immune response^29–34^. Additionally, it could be speculated that the migration of these cells from their bone marrow niches affects the hematopoiesis during sepsis.

For the first time, we have reported the mobilization of the developmentally primitive population of VSEL cells in septic shock patients. Two subsets of VSELs were distinguished, namely, CD34+ and CD133+ VSELs. Both subsets were found to migrate early to the blood circulation of septic shock patients. Notably, there was a negative correlation between Lin-CD133+CD45− VSELs and the PaO_2_/FiO_2_ ratio, suggesting the effects of hypoxia and lung injury on the mobilization of these cells. Thus, the observed mobilization of VSELs in septic shock with lung injury is in agreement with their potential to differentiate into alveolar epithelial cells^35^. Whether mobilized VSELs in sepsis do not remain dormant and induce differentiation pathways remains to be tested.

In agreement with the previous reports, we found early mobilization of cEPCs in septic patients^18,36,37^. Our results clearly show that the CD34+CD133+VEGFR2+ cells demonstrated a distinct pattern of circulation compared to HSPCs. Based on research performed on patients with chronic conditions, it is considered that the mobilization of cEPCs is a protective mechanism^38^.

We attempted to evaluate the prognostic value of circulating stem cells in septic shock. The ROC analysis revealed that the Lin-CD133+CD45+ HSCs and Lin-CD34+CD45− VSELs showed moderate accuracy in predicting mortality. Patients with greater numbers of those cells in circulation were less likely to survive. A similar relationship has been reported by Tsanagas *et al*.^26^ for CD34+CD45+ cells. Whether HSCs and VSELs could serve as biomarkers of sepsis requires further investigation in a dedicated prospective trial.

Finally, in order to understand the mechanism responsible for the observed differential mobilization of stem cells in sepsis, we conducted an *in vitro* analysis of the chemotactic activity of plasma from septic patients. Surprisingly, plasma from septic patients showed significantly less chemotactic activity on bone marrow nucleated cells as well as HSCs. This decreased activity of septic plasma was correlated with the lower concentration of S1P in septic patients, which is the major plasma chemoattractant for HSCs^39^. Additionally, we have shown that inhibition of S1P-S1P receptor signaling reduced migration of HSCs to normal plasma to the level comparable with the migration of HSCs to septic plasma. These observations support the involvement of S1P in the observed differences in mobilization of HSC in septic patients. Our results are in a striking contrast with observations of stem cell mobilization shortly after myocardial infarction, which showed an increase in S1P concentration followed by HSCs and VSELs mobilization^40^. In contrast, another important stem cell chemoattractant, CXCL12, was found to be elevated in septic plasma. Elevation of CXCL12 has been previously reported^36,37^, but in the context of the rest of our findings, it can be hypothesized that CXCL12 is a plasma chemoattractant for VSELs and cEPCs in sepsis but not for HSPCs. Indeed, CXCL12 has recently been shown to mobilize cEPCs in septic mice^41^. Our hypothesis is also supported by the finding that the cytokine milieu modulates the mobilization and retention of stem cells^42^.

Despite our efforts to perform a comprehensive analysis of the circulation of stem cells in septic shock patients, our study has several limitations. First, we did not conduct a functional analysis of the circulating progenitor cells or specific gene-expression assays. Instead, we focused on a rigorous flow cytometry analysis of different cell phenotypes, which allowed for simultaneous comparison of their circulation kinetics. Additionally, we measured the concentration of chemotactic mediators only on the day 1. The study protocol did not include comparison of septic shock patients with a group of non-septic critically ill patients which makes it impossible to answer whether the observed changes are specific to septic shock only or associate with critical illness. We are also aware that the studied group was relatively small, and a follow-up study to confirm the prognostic utility of stem cell evaluation in a new cohort of patients should be performed.

Experimental studies have shown that stem cell-based therapy may be beneficial in septic animals^43^. Whether the modulation of the autologous stem cells is beneficial in sepsis remains unknown, but we showed stem cell mobilization by the G-CSF in a septic shock patient^44^. Well-designed experimental studies are required to verify whether the enhancement of particular stem cell populations can provide benefits in sepsis.

## Conclusions

In conclusion, we observed a differential mobilization of stem cell subsets in sepsis related to changes in the levels of plasma chemoattractants. Early mobilization of primitive VSELs and cEPCs is a part of the stress response in sepsis. The mobilization of VSELs and early HSCs was found to be correlated with mortality. Moreover, sepsis was shown to strongly affect the population of HSCs, inducing their proliferation and mobilization. Whether autologous stem cells can become a novel and useful biomarker or therapeutic target in sepsis requires further study.

## Supplementary information

Online Supplement

## Data Availability

The datasets used and/or analyzed in this study are available from the corresponding author upon reasonable request.
