# Supplementary material for: Mobilization of Stem and Progenitor Cells in Septic Shock Patients
Source: Sci Rep. 2019 Mar 1;9:3289. doi: 10.1038/s41598-019-39772-4 (PMC6397313; doi:10.1038/s41598-019-39772-4)
Supplement: Supplementary file 1 — Online Supplement [file 41598_2019_39772_MOESM1_ESM.pdf]

Mobilization of Stem and Progenitor Cells in Septic Shock Patients

Tomasz Skirecki MD, PhD[1,2,3], Małgorzata Mikaszewska-Sokolewicz MD, PhD[4], Marlena Godlewska MSc, PhD[5], Barbara Dołęgowska[6], Jarosław Czubak MD, PhD[7], Grażyna Hoser MSc, PhD[2], Jerzy Kawiak MD, PhD, Prof[3], Urszula Zielińska-Borkowska MD, PhD[1]

Online data supplement

Supplemental Methods

Blood Collection

Five milliliters of peripheral blood (PB) was withdrawn via the central line catheter on Day 1, Day 3 and Day 7 after patient`s enrolment into the study. Serum was obtained immediately and stored in -80°C. Two milliliters of PB was taken out into EDTA-containing tube for flow cytometry (FACS) analysis and plasma separation. Also, five milliliters of peripheral blood were sampled via the venous puncture from healthy controls. After collection, the blood was immediately transported the Laboratory of Flow Cytometry.

S1P concentration

Serum and internal standard C17-S1P (Avanti Polar Lipids) used in this experiment were brought to room temperature. 100 µl of plasma/serum, 30 µl of synthetic C17-S1P standard and methanol (MetOH):10 mM K_2HPO_4 (9:1, v/v), at pH 7.2, were added to the glass tube. Under the same conditions a mixture containing C17-S1P and C18-S1P was prepared. Samples were vortexed and then 1M sodium chloride (NaCl) added to obtain 1ml. Subsequently, 1 ml of MetOH, 300 µl of concentrated hydrochloric acid and 2 ml of chloroform were added. Each step was preceded by mixing with a vortex. The samples were mixed on test tube rotator for 20 minutes and

centrifuged (3500 rpm, 20° C, 3 minutes). The lower organic phase was withdrawn and transferred to a new tube. The upper layer was re-extracted by adding 2 ml of chloroform, mixing on the test tube rotator for 10 minutes and re-centrifuging. The lower organic phase containing S1P was combined with the previous lower layer. The samples were then dried in a vacuum centrifuge (RVC 2-25 CD) at 45°C for about 45 to 60 minutes. The dried extracts were stored at -80°C until analysis. Before measurements, the extracts were brought to room temperature and dissolved in 130 µl methanol and 20 µl ortho-phthalaldehyde (OPA). Simultaneously, a mixture was prepared (prepared mix) consisting of 30 µl C17-S1P and 30 µl C18-S1P and 940 µl MetOH:K_2HPO_4 (9:1, v/v) at pH 7.2, from which 600 µl was taken, transferred to a new sample tube and 75 µl OPA added. All samples with OPA were incubated for 20 minutes at room temperature in a dark place and then centrifuged (6000 rpm, 20°C, 10 min). The supernatant was transferred to a new sample tube and 20 µl of 10mM K_2HPO_4 buffer at pH 7.2 was added. After centrifugation, the mixture was immediately transferred to a clean bottle. Buffer samples were incubated for 10 minutes at +4°C and then centrifuged again (6000 rpm, 20°C, 10 min). After centrifugation, the clear supernatant was transferred to a clean bottle and reversed-phase high-performance liquid chromatography analysis (RP-HPLC) was performed. Chromatographic data was developed using HP Chemstation software (Agilent, USA). C18-ARII Cosmosil 5 µm C18-ARII column (150 x 4.6) at 25°C and a 5 µm C18-ARII pre-column (10 x 4.6) (Waters) were used for separation in the reverse phase. An isocratic method with a mobile phase consisting of 10 mM K_2HPO_4 at pH 5.5 and methanol (15:85, v/v) was used. Samples of 50 µl were injected on the column every 30 minutes at a flow rate of 1 ml/min. The wavelength for detecting S1P derivatives was 340 nm for excitation and 455 nm for emission. S1P

concentration was calculated on the basis of the peak surface area of the internal standard C17-S1P.

Supplementary figure

Supplemental Figure 1. Flow cytometry analysis of the hematopoietic stem and progenitor cells. (A) First, cells of low granularity and size were gated. (B) Expression of CD34 versus CD38 was assessed then.

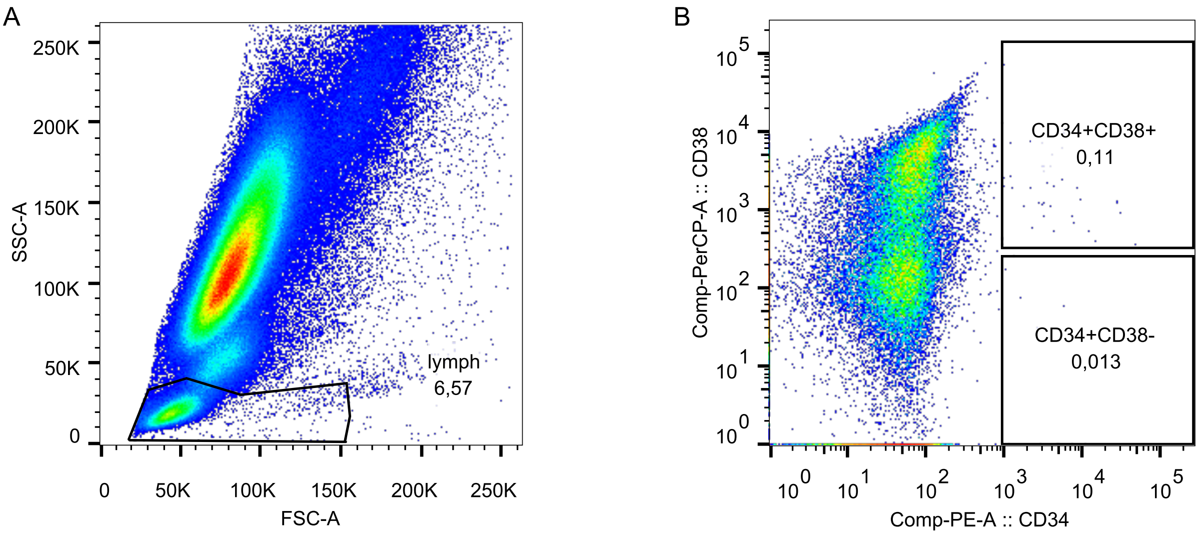

Supplementary Table 1. Panels of monoclonal antibodies used for the analysis of circulating stem cells in septic shock patients.

No	Antibodies panel (fluorophore, manufacturer, clone)	Assessed population
1	Lineage-FITC (BD; CD19 (HIB19), CD2 (RPA-2.10), CD3 (HI10a), CD4 (RPA-T4), CD14 (M5E2), CD66b (G10F5), CD24 (ML5), CD16 (3G8), CD56 (NCAM16.2), CD235a (GA-R2)); CD45-AmCyan (BD; 2D1); CD34-Pe.Cy7 (BD; 581); CD133-APC (Militenyi Biotec, Bergisch Gladbach Germany; AC133/1);	CD34+HSCs, CD34+VSELs, CD133+HSCs, CD133+VSELs
2	CD34- PE (BD; 581); CD38- Pe.Cy5 (BD; HB7)	CD34+CD38- HSCs, CD34+CD38+ HPCs
3	CD34-APC (BD; 581); Lineage-FITC; Ki-67-PE (BD; B56)	Proliferating HSPCs
4	CD34-PE (BD; 581); CD133-APC (Militenyi Biotec; AC133/1); VEGFR2-FITC (R&D Systems, Minneapolis, USA; 89106)	cEPCs

cEPCs: circulating endothelial progenitor cells; HPCs: hematopoietic progenitor cells; HSCs: hematopoietic stem cells; HSPCs: hematopoietic stem and progenitor cells; MSCs: mesenchymal stem cells

Supplementary Table 2. Percentage and total cell count values of circulating cells in the septic shock patients.

		Healthy Controls	Sepsis Patients D1	D1 *vs* C (p)	Sepsis Patients D3	D3 *vs* C (p)	D3 *vs* D1 (p)	Sepsis Patients D7	D7 *vs* C (p)	D7 *vs* D1 (p)
**Leukocytes**	**WBC (/ml)**	5.7x10^{6} (5.01x10^{6}-6.46x10^{6})	15.02x10^{6} (9.59x10^{6}-18.33x10^{6})	**<0.01**	17.58x10^{6} (12.6x10^{6}-21.68x10^{6})	**<0.01**	>0.05	10.59x10^{6} (8.25x10^{6}-16.03x10^{6})	**<0.01**	>0.05
**HPCs**	**CD34+CD38+ (%)**	0.028 (0.018-0.048)	0.0065 (0.0015-0.01)	**<0.01**	0.0195 (0.0044-0.0295)	>0.05	>0.05	0.0037 (0.008-0.018)	**<0.05**	>0.05
	**CD34+CD38+ (/ml)**	1615 (742-2630)	627 (217-2248)	>0.05	2106 (465-2626)	>0.05	>0.05	256 (61-1390)	>0.05	>0.05
**HSCs**	**CD34+CD38- (%)**	0.0035 (0.0019-0.055)	0.0007 (0.0004-0.0012)	**<0.05**	0.0052 (0.0024-0.0080)	>0.05	>0.05	0.0 (0.0-0.0003)	**<0.01**	>0.05
	**CD34+CD38- (/ml)**	169 (106-265)	81(14-408)	>0.05	507 (407-746)	**<0.01**	>0.05	0 (0-24)	**<0.05**	>0.05
**HSCs**	**Lin- CD34+ CD45+ (%)**	0.052 (0.025-0.12)	0.0093 (0.0021-0.025)	**<0.01**	0.0085 (0.0029-0.0130)	**<0.01**	>0.05	0.009 (0.002-0.012)	**<0.01**	>0.05
	**Lin- CD34+ CD45+ (/ml)**	3200 (1421-6000)	1707 (309-2477)	**<0.05**	555 (420-1903)	**<0.05**	>0.05	850 (251-1250)	**<0.01**	>0.05
**HSCs**	**Lin- CD133+ CD45+ (%)**	0.0025 (0.0012-0.0056)	0.0044 (0.00085-0.016)	>0.05	0.0055 (0.0013-0.0110)	>0.05	>0.05	0.0052 (0.0018-0.0066)	>0.05	>0.05
	**Lin-CD133+CD45+ (/ml)**	153 (65-188)	575 (172-1828)	**<0.01**	418 (275-1389)	**<0.01**	>0.05	430 (216-905)	**<0.05**	>0.05
**VSELs**	**Lin- CD34+ CD45- (%)**	0.0006 (0.00038-0.001)	0.001(0.0003-0.0045)	>0.05	0.0014 (0.0005-0.0041)	>0.05	>0.05	0.00057 (0.0003-0.0019)	>0.05	>0.05
	**Lin- CD34 CD45-+ (/ml)**	39 (17-46)	170 (46-455)	**<0.01**	264 (114-423)	**<0.01**	>0.05	67 (31-256)	>0.05	0.16
**VSELs**	**Lin- CD133+ CD45- (%)**	0.00049 (0-0.00076)	0.0002 (0-0.0027)	>0.05	0.0014 (0.0003-0.0035)	>0.05	>0.05	0.0013(0.0004-0.0044)	>0.05	0.5
	**Lin- CD133+CD45- (/ml)**	22 (0-43)	33 (0-161)	>0.05	167 (50-469)	**<0.01**	>0.05	175 (56-540)	**<0.05**	**0.8**
**cEPC**	**CD34+CD133+VEGFR2+ (%)**	0.0012(0.0009-0.0015)	0.0012(0.0004-0.0020)	>0.05	0.0015(0.0005-0.0020)	>0.05	>0.05	0.0011(0.0003-0.0016)	>0.05	>0.05
	**CD34+CD133+VEGFR2+ (/ml)**	58 (54-70)	236 (100-256)	**<0.05**	155 (106-209)	0.05	>0.05	175 (29-250)	>0.05	>0.05

C: healthy control group; cEPC: circulating endothelial progenitor; D1: Day 1; D2: Day 3; D7: Day 7; HPCs: hematopoietic progenitors; HSCs: hematopoietic stem cells; VSELs: very small embryonic-like stem cells; WBC: white blood count
